# Epidemic Erysipelas

**Published:** 1855-03

**Authors:** Wm. S. Marsh

**Affiliations:** West Point, Iowa


### EPIDEMIC ERYSIPELAS—ITS NATURE AND TREATMENT.

BY WM. S. MARSH, M. D., WEST POINT, IOWA.

Epidemic Erysipleas is a febrile disease, of an active grade, attended with diffusive inflammation, characterised by redness, burning heat, swelling, and vesication. The attack is usually preceded by slight headache, loss of appetite, fevered tongue, and an uneasy sensation in the epigastrium. After a short continuance of these symptoms, the patient is seized with a distinct rigor, violent pain in the head, back and extremities, difficult deglutition, with inflammation of the tonsils, and parotid, sub-maxillary, and sub-lingual glands; also of the sub-cutaneous and intermuscular cellular structure, assuming the character of a common phlegmonous inflammation. The pulse becomes hard, tense and frequent, and the tongue is often covered with a dark coat, and sometimes is extremely swollen.

The swelling of the tissues usually commences on the first day of the fever, and in the course of a few days small vesicles make their appearance on the inflamed surface. The disease terminates by one of three modes. The first, and perhaps the most frequent, if properly treated, is resolution, which occurs in from five to ten days. Frequently, however, the pain becomes throbbing, and at the same time the redness diminishes, and more or less extensive suppuration ensues. In still other cases, there is a gangrenous tendency, which may either be originally dependent upon the depressing nature or malignancy of the cause, upon the depraved condition of the system, especially of the blood, or upon certain co-operating influences of a debilitating character ; or, it may result from the excessive violence of the inflammation itself.

It is not unfrequently the case that there appears to be a complete metastasis of the disease to the viscera of the abdomen. When the disease occurs in this form, it is apt to assume a typhoid character, and is generally accompanied with low delirium throughout the greater part of its course, with a constant tendency to gangrene. This tendency is indicated by a peculiarly hot and burning pain, and a purple or livid hue of the surface. The near approach of the gangrene is shown by the slowness with which the blood returns, after removal by pressure, or by a doughy feel of the parts and the formation of small blisters, filled with a turbid or reddish serum.

Where the metastasis is referred to the lower extremities, it is generally attended with extensive suppuration, which takes place in from five to ten days, and is usually accompanied with irregular chills. In some cases the pus remains for a long time, before an opening is formed, to give it vent ; but when a natural or artificial orifice is made, the matter escapes, mingled with shreds of gangrenous cellular tissue. In these cases, the course of the disease is very tedious, and sinuses are often formed. The surface is tense, shining and usually pale. When pressed upon, it feels in some cases hard and resisting, but more frequently, it yields that peculiar sensation described by the term boggy. There is always most excruciating pain, of a burning and throbbing character. The pus burrows deep, sometimes inducing necrosis of bone. The pulse is always frequent, without strength and steadiness. The countenance is anxious and haggard. The mind is irritable and often delirious, and the patient frequently sinks, exhausted by the slow fever, and the extensive suppuration.

When the disease retains possession of the part at first attacked, it gradually spreads over a large extent of surface, and in some instances, when upon the head, the eyes are closed, and the whole face presents the appearance of a bladder distended with water. It is always more dangerous when it attacks the head, than when it occurs on the body, or the lower extremities. Instances occur in which the inflammation passes down into the fauces and gives rise to great dyspnœa.

I have attended cases, in which acute pneumonia came on during the hight of the disease, and one case in which for the space of two weeks, there was great dyspnœa, accompanied by violent and pungent pain in the right side of the chest, greatly increased by any attempt at a full inspiration or any coughing. The cough was short and dry, attended for some ten days with glairy and nearly colorless sputa, and stifled as much as possible, to avoid the great increase of pain which it occasioned. The respiration was performed chiefly by the diaphragm and abdominal muscles. These symptoms continued for ten days, at which time the pain gradually subsided, the cough became less painful and was attended by a copious expectoration not unlike that of a case of phthisis, in its later stages. The pulse became small and quick, there were hectic fever and night sweats. In short, the patient manifested every appearance of speedy dissolution.

But, after a few days' continuation of these very unfavorable symptoms, a gradual improvement was indicated, by the pulse becoming more slow, soft and full, expectoration less copious, respiration more natural and cough less troublesome. Her convalescence was more rapid than could have been expected. The sequel in this case, convinced me that a large abscess had formed on the pleura costalis.

As to the cause of epidemic erysipelas, nothing very definite can be said. Physicians of equal eminence and ability, come to different conclusions in their investigations. Some tell us that it depends upon a peculiar atmospheric condition, or miasm, while others contend that the disease is propogated by contagion. That there is some unknown condition of the atmosphere which greatly favors the production of this disease, I have no doubt, because, when this condition exists, the slightest influences, such as tend to depress or debilitate the system—as excessive fatigue, violent mental emotion—as of fear or anger and any undue excitement or irritation of the skin—as from the direct heat of the sun—will give rise to this form of inflammation; influences which, under other circumstances have no such effect. From the knowledge which I myself have had of the disease in question, I have no doubt of the fact, when we have typhus fever and erysipelas prevaling together of its communicability. Out of twenty-seven cases which I treated in one year, twenty could be traced to connection with the individual before referred to, who came to this town with the disease, having removed directly from Aurora, Indiana, where it was then extensively prevailing, attended by great mortality.

With respect to the prognosis, a sudden and marked alteration in the physiognomy, constant change of position, or lying continually in the same position, or, jactitation, succeeding to quietude in the later stages of this disease, are generally very unfavorable symptoms. The automatic movements, by which the patient seeks to carry his hand to his body, while the physician is examining his pulse, especially when accompanied by attempts to throw aside the bed clothes, and ineffectual efforts to rise, are also nearly fatal symptoms. The appearance of greyish or blackish spots beneath the separated epidemics, or a total suspension of the circulation in any part of the surface, with a reduction of the pain and a diminution of the temperature, are symptoms always indicative of great danger. A complete metastasis of the disease to the viscera of the abdomen, is generally accompanied by a constant tendency to gangrene, and a rapid dissolution. Colliquative diarrhœa, or muco-purulent discharges from the bowels, are also very unfavorable indications.

Respecting the general treatment of this disease, there exists as much discrepancy of opinion, as in regard to the proximate causes. As far as my own experience has enabled me to judge, my convictions are that in the majority of cases, the treatment for the first few days, should be strictly antiphlogistic. The indications for treatment are, first to diminish the inflammatory action and febrile excitement, then to correct the secretions and allay irritation, to arrest the extension of the disease, and to give free exit to all morbid secretions. If called within the first twenty-four hours, prompt venesection should be employed, to the degree if possible of producing syncope, opening a large orifice, with the patient erect, in order to produce the requisite effect by losing as little of the circulating fluid as possible. If deglutition has become difficult, as is usual in this affection, no time should be lost, during the relaxed condition of the patient's system caused by bleeding, in administering an emetic of ipecac, and wine of antimony. This has a tendency to equalize the circulation, and at the same time cleanse the stomach, dislodges the morbid secretions from the fauces and glottis, and acts as an efficient gargle. After the action of the emetic, a purgative may well be employed to correct the biliary derangement, which usually coexists.

℞ Protochl. Hydrarg.
Bicarb. Sodæ *aa* grs. x.
Ipecac pulv. " v. M

to be followed in three hours with Oleum Ricini. The regular action of the cutaneous exhalents should be supported by diaphoretics. Small portions of wine of antimony and spiritus ætheris nit., repeated every two hours answer a very good purpose. If the tongue is much swollen, as is sometimes the case, some three or four free incisions from two to two and a half inches long should be made upon its upper surface, and the patient should be directed to hold warm water in the mouth, to encourage the bleeding. Alterative portions of protochloride of mercury should be given from day to day, followed by castor oil, until there is a manifest improvement in the biliary secretions. If there should be no decided improvement by the third day, the emetic should be repeated. If there is an abundant secretion of a glairy, tough, tenacious character, free use should be made of a gargle composed of

℞ Acidi Nitrici

Aquæ fontanæ *aa* ℥ij

Capsici

Muriat Sodæ. *aa* ʒss M.

With respect to the local applications, we have, I think, nothing superior to the nitrate of silver, in solution, in proportion of ten grains to the ounce. Pieces of linen moistened with the solution should be laid on the inflamed surface, and renewed as occasion may require, until the inflammation begins to subside. Painting the sound skin in the form of a band, completely encircling the inflamed surface with the tincture of iodine, or cauterising with nit. silver, will often arrest the further progress of the disease. If there be manifest symptoms of gangrene having commenced, wine opium, camphor, quinine and the mineral acids should be freely given. The utmost caution should be observed, to prevent irritating the bowels, which is especially likely to induce metastasis.

Should extensive suppuration occur, opium, barks and quinine will be the best supporters of the system. When there is a sense of fluctuation, or when the skin is becoming livid or dusky, free incisions are absolutely necessary for the discharge of matter and slough. These are not merely apertures for the discharge of pus, but are very effectual means of cutting short the inflammation, by relieving the tension, and by evacuating the distended blood vessels. They should be carried quite deeply through the diseased tissues, and should be repeated as often as may be necessary.

If secondary inflammation occurs in some internal structure, cupping, sinapisms, blisters and the usual revulsives are indispensable.

Cases sometimes occur that in appearance closely resemble glossitis. Mr. G——, aged about fifty years, rather plethoric, and of a nervo-sanguine teneperament, was attacked with soreness of the throat, pain, swelling, inflammation of the tonsils, and parotid and submaxillary glands, difficult degultition and occasional chillness. He consulted his family physician, who, it would appear, was at a loss to make out the nature of the complaint. He, however, made a prescription for him, and advised him, in case he was not relieved by it, to send for me, being aware that I had treated a number of cases of what was then termed "black tongue." I was accordingly sent for, and saw him within twenty-four hours from the attack, and found him with the glands of the neck very much inflamed and swollen. The tongue presented the appearance of acute glossitis, being very much swollen, protruding between the teeth, quite painful and covered with a blackish coat. The fever was high, headache intense, deglutition entirely cut off, pulse ninety-five, full, strong and bounding. It was with the greatest difficulty that he could articulate, even in a low whisper. He was very anxious to ascertain my opinion of his case, and the treatment I intended to employ. I briefly informed him that his case was one of epidemic erysipelas, and that the treatment must be decidedly antiphlogistic; in short, that he must be bled to syncope. Being strongly prejudiced in favor of the botanic system, he strenuously objected to the treatment; but, when convinced that I would not undertake the case, short of carrying out the course suggested, he finally consented. I then requested him to assume the erect position, opened a vein in each arm, and suffered the blood to flow from large orifices, till syncope came on. He was then gently laid on a matrass, and the syncope continued as long as I dared to prolong it.

The effect was like a charm—the perspiration stood at every pore—the spasm about the glottis gave way—and he was enabled to swallow. This truce was taken advantage of, and an emetic of antimonial wine was administered, and small doses continued from time to time, in order to protract the vomiting as long as was safe. I then ordered a purgative of protochloride of mercury, bicarbonate of soda, and ipecac, in the proportions previously mentioned, to be followed in three hours with ℥i ol. ricini; also a diaphoretic mixture composed of

℞ Vin. Antimonii

Sps. Ætheris Nitr. *aa* ℥i

Ipecac grs. xv

Aquæ font. ℥iv M

to be given in ʒij doses every two hours through the night.

Second day, 8 o'clock a. m.—Pulse ninety; more soft and compressible; skin less hot, and moist; cathartic had operated freely; less pain in the head; swelling of the glands stationary; difficulty of deglutition returning; tongue more swollen, and protruding far between the teeth. Knowing that something must be done to remove the congestion in this organ, I determined to scarify, and accordingly passed the blade of a bistoury upon the dorsum of the tongue as far back as it could conveniently be done. It was then turned upon its edge, and withdrawn, making a deep incision into the substance of the tongue. Two or three other incisions were made in the same manner, and the patient ordered to hold warm water, as previously suggested. The free use of Tinct. Iodine was ordered as an external application to the surface of the neck. The diaphoretic mixture was continued with five grains of Iodine of Potassium every six hours.

Third day, 10 o'clock, a. m.—Pulse 85, soft and full; the swelling of the tongue and glands disappearing; skin soft and moist; patient annoyed much with a tough and viscid mucus about the glottis, which he has great difficulty in dislodging. Ordered the gargle before alluded to, which was used every hour. Bowels to be moved with castor oil and spirits of turpentine; Iodide of Potassium to be continued and the Tincture of Iodine externally.

Fourth day, 2 o'clock p. m.—Patient nearly well; pulse seventy-five; skin moist and of natural temperature. Ordered the continuation of the Iodine for a few days; the patient to be confined to an even atmospheric temperature, using Sps. of Mindereri and mild laxatives; subsisting on a mild unirritating diet, and employing freely the tepid bath, strictly avoiding everything calculated to induce a relapse. Dismissed the patient; no unfavorable symptoms occurred after this time.

Out of the twenty-seven cases thus treated, twenty-five recovered; the other two cases appeared at first so mild as not to justify the antiphlogistic treatment and died of metastasis to the viscera of the abdomen, producing rapid dissolution.

We must never forget, however, that the disease varies very much in its type, at different periods, sometimes requiring decided antiphlogistic measures, and at other times, an opposite course of treatment.

